# Changes in Pulse Rate, Respiratory Rate and Rectal Temperature in Working Dogs before and after Three Different Field Trials

**DOI:** 10.3390/ani10040733

**Published:** 2020-04-23

**Authors:** Mirella Lopedote, Simona Valentini, Vincenzo Musella, Jose Manuel Vilar, Giuseppe Spinella

**Affiliations:** 1 Clinica Veterinaria San Michele, 38010 Grumo di San Michele all’Adige (TN), Italy; mirella.lopedote@yahoo.com; 2 Department of Veterinary Medical Sciences, University of Bologna, 40064 Ozzano dell’Emilia (BO), Italy; simona.valentini@unibo.it (S.V.); giuseppe.spinella@unibo.it (G.S.); 3 Department of Health Sciences, University of Catanzaro, 88100 Germaneto (CZ), Italy; musella@unicz.it; 4 Department of Animal Pathology, Instituto Universitario de Investigaciones Biomédicas y Universitarias, Universidad de Las Palmas de Gran Canaria, 35416 Arucas, Spain

**Keywords:** pulse rate, respiratory rate, rectal temperature, working dogs

## Abstract

**Simple Summary:**

Competitions dedicated to working dogs have greatly expanded in order to select the best canine individuals for Search-and-Rescue (SAR) activity. Therefore, it is essential to identify physiological parameters for a rapid clinical evaluation in order to provide useful information on the physical fitness of athlete dogs. In the present study, heart rate, respiratory rate and rectal temperature were investigated during three different working activities. Heart rate was the most conditioned parameter by exercise and, probably, the most useful to evaluate the canine predisposition and response to work. Respiratory rate and rectal temperature were more affected by environmental variables.

**Abstract:**

Physiological changes (pulse rate, respiratory rate and rectal temperature) induced by exercise are usually studied as physical fitness indices. The aim of this study was to investigate how these physiological parameters could be modified in a group of trained working dogs during three different field trials (rubble, search on field, obedience), in order to assess which parameter would be more useful to detect the dog response to exercise. Nine dogs were included in this study. The animals were monitored at rest, immediately before and after the working session. Pulse rate values increased significantly in all the phases compared to rest status. Respiratory rate values increased significantly after the competition, while rectal temperature was significantly increased only after search on rubbles and obedience activities. Reference values for specific competitions need to be stablished in order to promptly identify poor performance or exercise intolerance.

## 1. Introduction

Search and rescue dogs have a great social impact due to the valuable help in finding survivors during catastrophic events, such as earthquakes [1]. Over the last years, competitions dedicated to working dogs have greatly expanded in order to select the best canine individuals for Search-and-Rescue (SAR) activity. Although skills such as obedience, bonding with the handler and detecting human smell are particularly important in this category [2,3], these athlete dogs need adequate physical fitness to cover wide areas of land when searching, even in adverse conditions [4]. Physical fitness was defined as “the body’s ability to maintain internal physiological balance as close as possible to the state of rest during physical exertion and to promptly restore altered balance after exercise” [5,6]. Physiological changes induced by exercise can therefore be studied as physical “fitness indices”. The physiological parameters frequently used for this type of investigation are: pulse rate, respiratory rate and rectal temperature [7,8]. This clinical evaluation can be followed by laboratory monitoring on plasma or serum for the early detection of muscle damage or excessive fatigue after work, although it is a more invasive method and the results could be mostly non-specific [3,7].

The aim of this study was to investigate the effects of exercise on pulse rate (PR), respiratory rate (RR) and rectal temperature (RT) in a group of Italian SAR dogs involved in three different field trials (rubble, search on field, obedience), in order to realize which parameter could be more useful to detect the dog’s response to exercise. Our hypothesis was that the three parameters could be differently altered depending on the typology of exercise performed by the dogs and by environmental changes.

## 2. Materials and Methods 

### 2.1. Animals

Nine healthy working dogs of different breeds were included in this study. Sound status was evaluated one day before the specific activity by a licensed Doctor in Veterinary Medicine. All animals were kept under a similar training approach (4 days per week) and fed with a commercial dog food for adult dogs. 

For this study, three working sessions (search on field, search on rubbles and obedience) were performed during the spring season (Central-Southern Italy, April 2019) within 24 h (from 2 p.m. of Saturday to 2 p.m. of Sunday), with a minimum rest time of two hours between different boots. 

**Figure 1:**
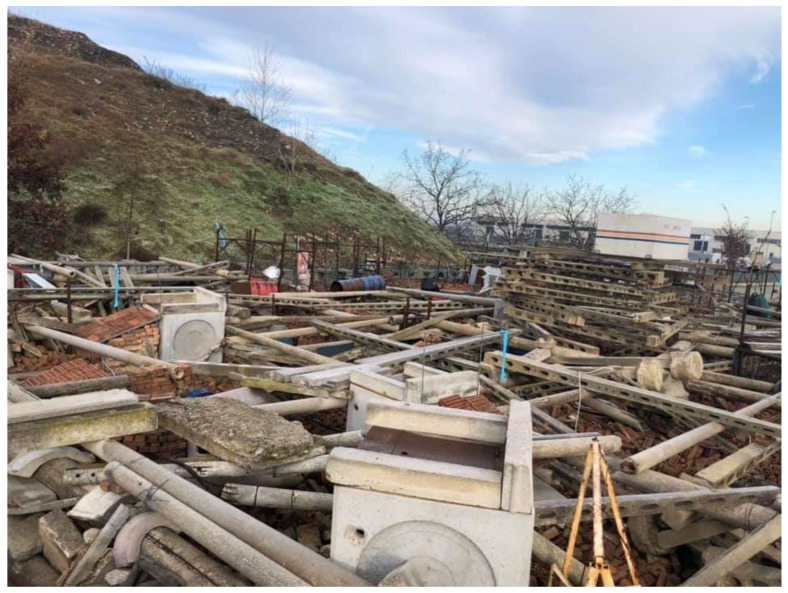
Scenario related to the rubble activity; specifically, the part with highly unstable concrete beams that reproduce the “*Shanghai game*” effect.

Each dog performed search on field for about twenty minutes on a two hectares area including 15 m high knolls, formed by stabilized sandy material and heaps of rubble and cement, large mud puddles, muddy and sandy soil. Dogs had to find or signal two “walk-ons simulating victims”. The search on rubble lasted maximum 20 min and, also in this step, two walk-ons should have been tracked by the dog. Part of the rubble was structured like a labyrinth, where the dog had to pass between pipes and nets on an unstable red brick surface to reach the walk-on. The other part developed vertically with highly unstable concrete beams with an effect similar to the “*Shanghai game*” (Figure 1).

The obedience activity consisted of walking with handler, walk and stop, ability of correct indifference to the presence of other dogs and walk around them, and indifference to particularly loud signals. This specific activity lasted a maximum of 15 min.

RR, PR and RT were monitored for each dog at rest (at home 24 h before competition), and before (within 5 min before activity) and after the working session (within 5 min). Environmental temperature and humidity were also recorded. 

Ethical approval for this study was obtained by Animal Welfare Committee of University of Bologna, in accordance with Italian DL 26/2014 (Project ID 914).

### 2.2. Statistical Analysis

All data were submitted to descriptive (mean ± standard deviation, and median) and analytic statistical analysis. For all variables by ANOVA, a post-hoc Bonferroni test was applied, in order to investigate any significant variation between rest before and after the three different working competitions. Significance was set at *p* ≤ 0.05. Statistical analyses were performed using the software: Stata version 15 (StataCorp, College Station, TX, USA, 2017).

## 3. Results

The included nine dogs were five Labrador, two Malinois, one German Shepherd and one mixed breed dog. They were six intact females and three males (two intact and one castrated), aged between 1 and 9 years (mean age 3.3 years). 

At rest, PR showed a mean value of 54.8 ± 9.31 beats per minute (median 56), mean RR of 26 ± 7.21 breaths per minutes (median 26) and mean RT of 38.3 °C ± 0.08 (median 38.5 °C). 

Recorded data related to environmental temperature and humidity, respectively, reported: 8 °C and 60% during rubble activity, 14 °C and 90% during search on field activity and 10 °C and 98% during the obedience competition. 

All data related to PR, RR and RT at rest, before and after the different trials are reported in Table 1. 

**Figure 2:**
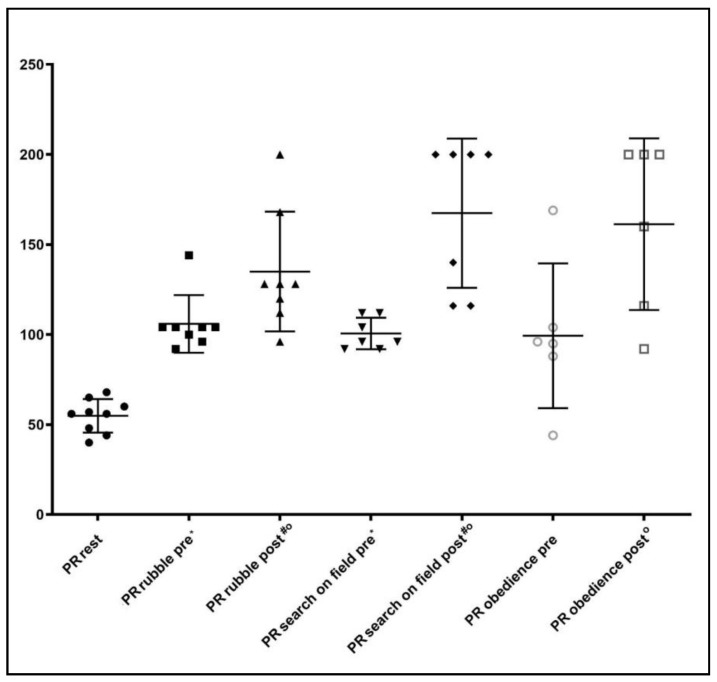
Pulse rate (PR) data obtained at rest, before (pre) and after (post) the specific activity. * = *p* < 0.05 between rest and before activity; ^#^ = *p* < 0.05 between before and after activity; ° = *p* < 0.05 between rest and post activity.

PR values increased significantly in all the phases compared to rest status (*p* < 0.05), except for obedience (*p* = 0.06) in the pre-activity phase (Figure 2). 

**Figure 3:**
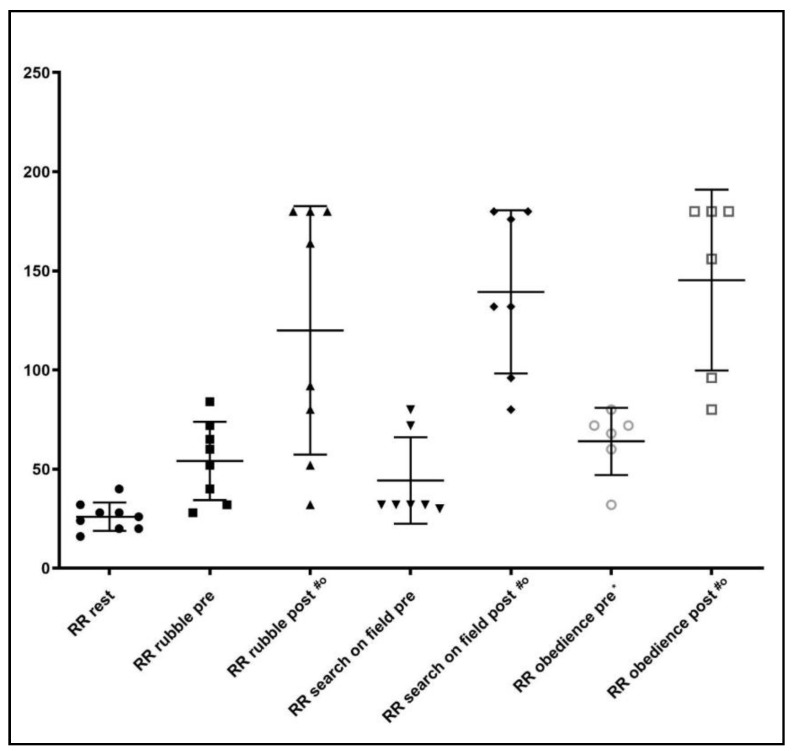
Respiratory rate (RR) observed at rest, before (pre) and after (post) the specific activity. * = *p* < 0.05 between rest and before activity (*p* < 0.05); ^#^ = *p* < 0.05 between before and after activity (*p* < 0.05); ° = *p* < 0.05 between rest and post activity.

RR values increased significantly after the competition (*p* < 0.05), while in the pre-competition phase they were similar to those at resting phase. Only in the obedience trial, an increased RR was observed before the working session (Figure 3).

**Figure 4:**
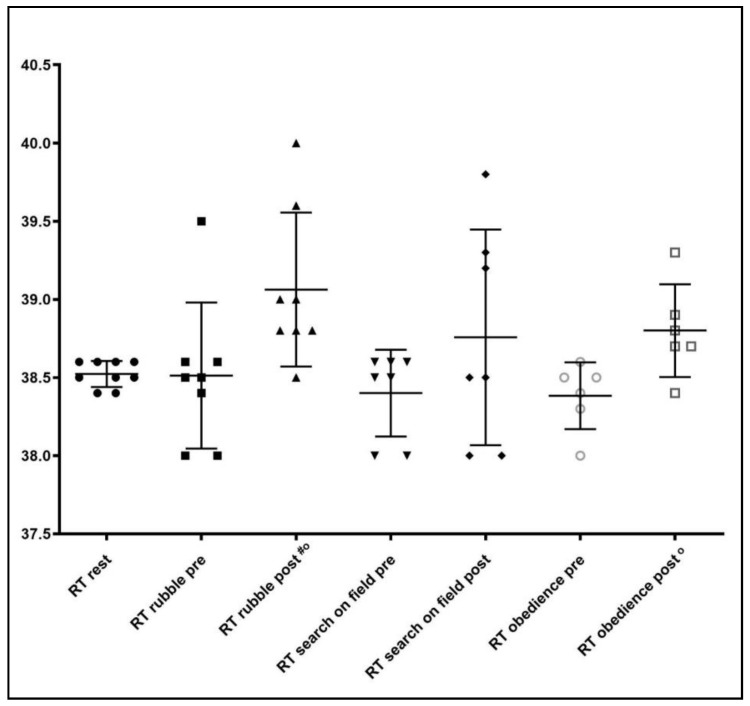
Rectal Temperature (RT) observed at rest, before (pre) and after (post) the specific activity. ^#^ = *p* < 0.05 before and after activity; ° = *p* < 0.05 between rest and post activity.

RT was significantly increased only after the search on rubbles (*p* = 0.02) and obedience activities (*p* = 0.006) (Figure 4).

## 4. Discussion

The aim of this study was to investigate variations of PR, RR and RT immediately before and after three different competitions compared to rest time. Variations of the three parameters were observed during the three detection times according to the working session.

PR was the only parameter that significantly increased in the pre-competition phases of search on rubble and open field, reflecting an anticipatory response to the competition-related excitement and to exercise intensity, regardless of environmental conditions. 

This physiological response was probably due to the adrenaline secretion, which anticipates intensive physical activity, through activation of the sympathetic nervous system and the hypothalamic-pituitary-adrenal axis by hormone cortisol [3]. This emotional response is also known as the “Eureka effect” [9]. An anticipatory increase in cortisol before sport competition is fundamental to prepare the athlete for the psychological and physiological demands, and it has been suggested that it highly affects sport performance through its influence on cognitive processes [10]. As a noninvasive cardiovascular marker, heart rate variability reflects the changes of the sympatho-parasympathetic balance of the autonomic nervous system (ANS) in response to external stimuli, as an indicator of stress and animal welfare [11]. PR variability was largely investigated as relevant information of ANS’s short-term response in horses and, in lesser extent, in dogs [7,12]; moreover, a meta-analytic study performed in human sports medicine has reported a significant increase in cognitive and somatic anxiety in the time leading up to competition. Cortisol concentrations during moderate intensive sports, such as activity performed by working dogs, lead to positive effects on the reaction time and inhibition of aversive stimuli [10]. The same meta-analysis has also underlined that cortisol release before competition did not change between gender (males and females) or between individual or team sports [10]. Otherwise, the level of competition could partially influence the cortisol release, because emotional response before sport competition was significantly lower in more experienced athletes, compared to those less experienced [10].

Compared to PR findings, RR and RT were apparently influenced by physical exertion and environmental parameters. Indeed, significant increases of RR or RT were not observed in pre-activity phases compared to rest, except for obedience activity, in which higher relative humidity was recorded. In particular, RR showed a significant increase after all three different activities compared to the rest and pre-competition phases. In our opinion, this observation should be mainly related to physical fatigue immediately after the competition, rather than environmental temperature and humidity. Only in the obedience trial, a significant increase of RR was revealed before the working session; this observation could be primarily due to the higher relative humidity (98%) recorded in that specific session. 

Breathing rate increases during and immediately after exercise to compensate the increased need of oxygen that is required to release energy. Exhalation leads to expel carbon dioxide and waste products of respiration; moreover, during exercise, lungs and respiratory system provide more oxygen to the blood and muscles to guarantee the oxidative muscular metabolism in these specific activities of medium duration and intensity. Moreover, hyperventilation can be also induced by excitement, metabolic acidosis, and stimulation of brainstem respiratory centers [8]. Our data demonstrated that environmental temperature and humidity primarily affect the respiratory apparatus, and only minimally the RT, as previously reported by other authors [7]. This limited effect on body temperature could be partially due to heat lost by evaporation observed during breathing activity. The elevation in rectal and muscle temperature resulting from prolonged exercise by dogs is associated with reduced levels of high energy phosphates (ATP and CrP) and increased levels of muscle lactate, pyruvate, and AMP, which may contribute to fatigue [13]. In greyhounds a limited, but positive, association between ambient temperature and post-exercise body temperature has been observed [14]. Those authors reported that when ambient temperature reached 38 °C, 39% of the included dogs showed a RT > 41.5 °C. The mean increase in rectal temperature was greater in dark than light colored greyhounds, and, in the post-race observation, in males than in females. In dogs, when the environmental temperature exceeds the body temperature, heat can only be lost by evaporation through the respiratory tract [14,15]. During strenuous exercise, the respiratory rate increases, but high levels of humidity may restrict the amount of heat lost. Another recent study did not find any direct correlation between ambient conditions and the post-race body temperature of dogs competing in canicross races. However, their results suggested that male dogs, dark coateddogs, and increased speed of running could represent a risk of heatstroke [16]. Greyhounds following even short periods of strenuous exercise commonly exhibit cramps and muscular fatigue [17]. However, susceptibility may vary between breeds of dog and, above all, on basis of specific exercise and duration of activity. No rectal temperature effects were observed in exercising dogs when the environmental temperature ranged between 11 °C and 28 °C [8]. In contrast, a significant association between ambient and rectal temperatures is present in sled dogs working in ambient temperatures between −9 and 25 °C [18]. Our data related to rectal temperature show a high analogy with the results of the study by Matwichuck et al. [8] and Steiss et al. [7], confirming that this parameter is not affected by the pre-race excitement or the environment, if dogs deal with medium duration and medium intensity exercises.

## 5. Conclusions

The results of our study suggested that PR increases in working dogs were largely due to the anticipatory response to the competition-related excitement in the pre-activity phase, and to physical effort in post-activity phase. RR variations in the post-activity phase were mainly affected by physical effort, and, partially, by environmental temperature and relative humidity. RT does not significantly alter in the pre-activity phase, and only minimally in the post-activity phase, probably in relation to environmental temperature and relative humidity. In conclusion, to avoid the sympathetic response, we suggest comparing all parameters just before and after excercise. However, further research is needed to confirm these preliminary conclusions and reference values for specific competitions, which need to be established in order to promptly identify poor performance or exercise intolerance.

## Figures and Tables

**Table 1:** Mean ± SD (median) of PR and RR expressed in beats and breaths per minute and RT expressed in °C before and after the three different activities.

	Rest	Before Rubble	After Rubble	Before Search on Field	After Search on Field	Before Obedience	After Obedience
PR	54.8 ± 9.31 (56)	106 ± 16 (104) ^a^	135 ± 33.24 (128) ^b,c^	100.57 ± 8.77 (96) ^a^	167.42 ± 41.40 (200) ^b,c^	99.33 ± 36.73 (95.5)	161.33 ± 43.49 (180) ^c^
RR	26 ± 7.21 (26)	54.12 ± 19.78 (56)	120 ± 62.66 (128) ^b,c^	44.28 ± 21.79 (32)	139.42 ± 41.14 (132) ^b,c^	64 ± 16.97 (70) ^a^	145.4 ± 45.65 (168) ^b,c^
RT	38.3 ± 0.08 (38.5)	38.5 ± 0.46 (38.5)	39 ± 0.49 (38.9) ^b,c^	38.4 ± 0.27 (38.5)	38.75 ± 0.69 (38.5)	38.3 ± 0.21 (38.45)	38.8 ± 0.29 (38.75) ^b^

^a^ = Statistical significance between Rest and Before activity; ^b^ = statistical significance between Before and After Activity; ^c^ = Statistical significance difference between Rest and After activity.
